# Chemometric Comparison and Classification of Some Essential Oils Extracted from Plants Belonging to Apiaceae and Lamiaceae Families Based on Their Chemical Composition and Biological Activities

**DOI:** 10.3390/molecules23092261

**Published:** 2018-09-05

**Authors:** Cristina Anamaria Semeniuc, Maria-Ioana Socaciu, Sonia Ancuţa Socaci, Vlad Mureșan, Melinda Fogarasi, Ancuţa Mihaela Rotar

**Affiliations:** 1 Department of Food Engineering, University of Agricultural Sciences and Veterinary Medicine Cluj-Napoca, 3-5 Mănăştur St., 400372 Cluj-Napoca, Romania; melinda.nagy@usamvcluj.ro; 2 Department of Food Science, University of Agricultural Sciences and Veterinary Medicine Cluj-Napoca, 3-5 Mănăştur St., 400372 Cluj-Napoca, Romania; maria-ioana.socaciu@usamvcluj.ro (M.-I.S.); sonia.socaci@usamvcluj.ro (S.A.S.); anca.rotar@usamvcluj.ro (A.M.R.)

**Keywords:** essential oils, chemical composition, total phenolic content, antioxidant activity, antibacterial activity, chemometrics

## Abstract

This study is focused on the comparison and classification of parsley, lovage, basil, and thyme essential oils (EOs) based on their chemical composition, total phenolic content, antioxidant and antibacterial activities by using appropriate chemometric methods: Principal component analysis (PCA) and hierarchical cluster analysis (HCA). The results showed that parsley, lovage, and thyme EOs are rich in monoterpene hydrocarbons, but basil EO is rich in oxygenated monoterpenes and phenylpropanoids, and that both PCA and HCA separated essential oils into two main groups of which one contains two sub-groups. β-Phellandrene was the major component identified in parsley and lovage EOs, estragole was the major component in basil EO, and *p*-cymene was the major component in thyme EO. Thyme EO showed the highest level of total phenolics, the highest antioxidant capacity, and exhibited the stronger antibacterial activity, results that were emphasized by both chemometric methods used. Among tested essential oils, the one of parsley was distinguished by a low total TPC, weak antioxidant activity, and weak antibacterial activity against *S. enteritidis* (ATCC 13076); lovage EO by low TPC, weak antioxidant activity, but moderate antibacterial activity; and basil EO by low TPC, moderate antioxidant activity, and weak antibacterial activity against *L. monocytogenes* (ATCC 19114).

## 1. Introduction

Essential oils are aromatic and volatile liquids distilled from different plant materials [1]. Numerous studies have shown that essential oils possess antioxidant, antibacterial, antifungal, and nematicidal activities, with their chemical compounds being responsible for these properties [2,3,4,5,6,7,8,9,10,11,12,13]. Plant essential oils constituents are mainly grouped in two distinct chemical classes: Terpenoids (monoterpene hydrocarbons, sesquiterpene hydrocarbons, and their oxygenated derivatives) and phenylpropanoids (phenols and phenol ethers) [14]. Both terpenoid and phenylpropanoid families comprise phenolic compounds which are considered to be mainly responsible for the antioxidant activity of essential oils [5,15]. In general, the climate conditions, geographical region, and several other factors directly influence the relative composition of essential oils [16].

Due to negative perceptions of synthetic preservatives among consumers, the interest in essential oils focusing on their application in food preservation has grown considerably in the last few years [1]. Essential oils added to edible products, either by direct mixing or in edible coatings and active packaging may stand as a valid alternative to prolong shelf-life and prevent autoxidation [15]. For this purpose, the food industry requires large amounts of essential oils. Vegetable raw materials (mainly leaves) used for large-scale extraction of essential oils are usually purchased from different suppliers. In their dry state they are more stable during storage and are therefore available throughout the year.

Our previous study [8] has shown that essential oils extracted from dried leaves of parsley, lovage, basil, and thyme possess antimicrobial properties. For a better understanding of their active compounds and mechanisms of action, a further in-depth study is required. Consequently, the present work aimed to determine any relationship between the chemical constituents, total phenolic content, antioxidant capacity, and antibacterial activity of parsley, lovage, basil, and thyme essential oils (EOs) extracted from dried leaves, in order to highlight the best potential of each one for future applications in the food industry. To the best of our knowledge, this is the first study that illustrates the strengths and weaknesses of these essential oils regarding their biological activities by using chemometric methods.

## 2. Results

### 2.1. Yield and Chemical Composition of Essential Oils

The highest essential oil concentration, as calculated on the basis of dry plant weight (*v*/*w*), was recorded in leaves of thyme (2.20%, *v*/*w*), followed by basil (0.40%, *v*/*w*), lovage (0.28%, *v*/*w*), and parsley (0.16%, *v*/*w*). The volatile compounds detected by ITEX-GC/MS analysis in the four essential oils with their percentage composition are summarized in Table 1.

Parsley essential oil: Twenty-three compounds, representing 99.8% of the total detected constituents, were identified in the essential oil of parsley and grouped on the basis of their chemical structure into six classes (monoterpene hydrocarbons-C1, sesquiterpene hydrocarbons-C2, oxygenated monoterpenes-C3, phenylpropanoids-C4, other compounds-C5, and unidentified compounds-C6). The most abundant constituents were monoterpene hydrocarbons (97.2%), followed by oxygenated monoterpenes (0.9%), phenylpropanoids (0.8%), others (0.5%), sesquiterpene hydrocarbons (0.4%), and unidentified compounds (0.2%). The major components identified in parsley EO were β-phellandrene (32.4%), 1,3,8-*p*-menthatriene (13.9%), 1,8-cineole (12.5%), β-myrcene (12.7%), α-pinene (6.5%), and 2,6-dimethylstyrene (6.4%), all of them belonging to the monoterpene hydrocarbons class. 1,3,8-*p*-Menthatriene is a characteristic volatile constituent of parsley leaves that contribute to the parsley aroma [17]. A similar aromatic profile was found by Petropoulos et al., 2004 [18] in essential oil extracted from dried leaves of Greek parsley at the first sowing and growth stage with β-phellandrene, 1,3,8-*p*-menthatriene, β-myrcene, and α,*p*-dimethylstyrene being the major components. In a study on essential oil from dried leaves of Estonian parsley, Vokk et al., 2011 [19] have noticed myristicin to be the major constituent, followed by β-phellandrene, 1,3,8-*p*-menthatriene, and β-myrcene.

Lovage essential oil: Eighteen compounds were identified in the essential oil of lovage representing 100% of the total detected constituents. The most abundant constituents were monoterpene hydrocarbons (83.6%), followed by oxygenated monoterpenes (15.6%), others (0.5%), and phenylpropanoids (0.3%). The major components identified in lovage EO were β-phellandrene (53.9%), β-terpinyl acetate (12.7%), β-myrcene (11.4%), and trans-β-ocimene (7.0%); the β-terpinyl acetate belongs to the oxygenated monoterpenes class and the rest of them to monoterpene hydrocarbons. The study of Bylaiteù et al., 1998 [20] has evidenced α-terpinyl acetate as the major compound of essential oil extracted from dried leaves of Lithuanian lovage, followed by β-phellandrene, and Z-ligustilide.

Basil essential oil: Twenty-four compounds were identified in the essential oil of basil representing 99.9% of the total detected constituents. The most abundant constituents were oxygenated monoterpenes (46.4%), followed by phenylpropanoids (41.2%), monoterpene hydrocarbons (10.7%), sesquiterpene hydrocarbons (1.6%), and unidentified compounds (0.1%). The major components identified in basil EO were estragole (40.9%), 1,8-cineole (24.3%), and linalool acetate (19.8%), first of them belonging to phenylpropanoids and the others to oxygenated monoterpenes. In a study on essential oil extracted from dried leaves of Polish basil, Calín-Sánchez et al., 2012 [21] have found phenylpropanoids as the majority class, followed by monoterpenoids, sesquiterpenes, monoterpenes, sesquiterpenoids, aldehydes, and alcohols with methyleugenol, eugenol, 1,8-cineole, and linalool being the major components.

Thyme essential oil: Nineteen compounds were identified in the essential oil of thyme representing 96.5% of the total detected constituents. The most abundant constituents were monoterpene hydrocarbons (83.5%), followed by oxygenated monoterpenes (9.7%), unidentified compounds (3.5%), others (3.0%), sesquiterpene hydrocarbons (0.2%), and phenylpropanoids (0.2%). The major components identified in thyme EO were *p*-cymene (49.8%), γ-terpinene (18.6%), thymol (8.7%), and β-myrcene (5.4%); the thymol belongs to the oxygenated monoterpenes class and the rest of them to monoterpene hydrocarbons.

A number of studies have focused on the chemical composition of essential oil extracted from dried leaves of thyme. Contrary to our study, Jamali et al., 2013 [22] have found oxygenated monoterpenes as the main constituents in Moroccan essential oil, followed by monoterpene hydrocarbons, sesquiterpene hydrocarbons, and oxygenated sesquiterpenes; the major components identified were carvacrol, γ-terpinene, *p*-cymene, and α-pinene. Borugă et al., 2014 [4] have identified thymol, γ-terpinene, and *p*-cymene as the major components of essential oil from thyme cultivated in Romania. In another study, Calín-Sánchez et al., 2013 [23] have identified thymol, γ-terpinene, *p*-cymene, caryophyllene, and α-terpinene as the major constituents of Polish essential oil. Agili, 2014 [3] has reported thymol, γ-terpinene, *p*-cymene, and carvacrol as the major constituents of essential oil extracted from thyme collected from Saudi Arabian markets. The main constituents found by Soto-Mendívil et al., 2006 [24] in essential oil extracted from thyme collected from Mexican markets were borneol, thymol, carvacrol methyl ether, camphene, α-humulene, and carvacrol.

### 2.2. Total Phenolic Content and Antioxidant Activity of Essential Oils

Table 2 shows the values for total phenolic content (TPC) and Trolox equivalent antioxidant capacity (TEAC) of essential oils. The highest TPC was found in thyme EO (22.5 ± 1.69 mg GAE 100 µL^−1^ EO), followed by essential oil of lovage (3.0 ± 0.15 mg GAE 100 µL^−1^ EO), basil (2.9 ± 0.13 mg GAE 100 µL^−1^ EO), and parsley (2.2 ± 0.05 mg GAE 100 µL^−1^ EO). Phenolics are a class of compounds that act as free radical scavengers and are responsible for the antioxidant activity of essential oils [2,5]. The highest antioxidant capacity was noticed in essential oil extracted from thyme (197.0 ± 0.23 µM TE mL^−1^ EO), followed by the one of basil (105.0 ± 1.09 µM TE mL^−1^ EO), parsley (39.4 ± 0.02 µM TE mL^−1^ EO), and lovage (35.9 ± 0.27 µM TE mL^−1^ EO).

It is well known that phenolic compounds act as antioxidants [25,26], but they are not fully responsible for the antioxidant activity of essential oils. In a recent study, Ferreira et al., 2017 [7] have reported an enhanced antioxidant activity of essential oils rich in oxygenated monoterpenes. In the current study, the level of total phenolics in basil EO was proximate to that found in parsley and lovage EOs; however, its antioxidant activity was approximately 3 times higher. Since oxygenated monoterpenes (46.4%) prevailed in basil EO, we first suspected that this class of compounds is the major contributor to its antioxidant activity. However, no significant correlation between TEAC and oxygenated monoterpenes-C3 was found. In contrast, there was a positive correlation (r = 0.999; *p* < 0.05) between TEAC and phenylpropanoids-C4. These findings show that phenylpropanoids (41.2%) are in fact mainly responsible for the antioxidant activity of basil EO.

### 2.3. Antibacterial Activity of Essential Oils

Table 3 summarizes the results of minimum inhibitory concentration and minimum bactericidal concentration tests. There was a strong positive correlation (r = 0.976; *p* < 0.0001) between values obtained using the two tests since the minimum inhibitory concentrations (MICs) and minimum bactericidal concentrations (MBCs) were similar, except those of parsley EO against *L. monocytogenes*. The lower the MIC/MBC value, the higher the antibacterial activity of an essential oil. Of the four essential oils tested, basil EO has proved to be more effective against *S. enteritidis* than *L. monocytogenes* and the others conversely. The results show that thyme EO was the most bacteriostatic/bactericidal against *S. enteritidis*, followed equally by lovage and basil EOs, and then by parsley EO. Thyme EO was also the most bacteriostatic/bactericidal against *L. monocytogenes*, followed instead by essential oils of lovage, parsley, and basil. The essential oil of parsley showed similar MIC and MBC values against *S. enteritidis* (22.68 µL EO mL^−1^); a MIC value of 5.14 µL EO mL^−1^ and MBC value of 10.80 µL EO mL^−1^ against *L. monocytogenes*. Essential oils of lovage, basil, and thyme revealed similar bacteriostatic and bactericidal activities against *S. enteritidis* (10.80 µL EO mL^−1^, 10.80 µL EO mL^−1^ and 0.56 µL EO mL^−1^, respectively) and *L. monocytogenes* (2.45 µL EO mL^−1^, 22.68 µL EO mL^−1^ and 0.13 µL EO mL^−1^, respectively). Jamali et al., 2013 [22] have reported levels of 270 µg EO mL^−1^ for MIC and 540 µg EO mL^−1^ for MBC in essential oil extracted from dried leaves of thyme tested against *L. monocytogenes*; an antibacterial activity much lower than the one found in the current study.

## 3. Discussion

### Comparison and Classification of Essential Oils Based on Their Chemical Composition, Total Phenolic Content, Antioxidant and Antibacterial Activities

**Figure 1:**
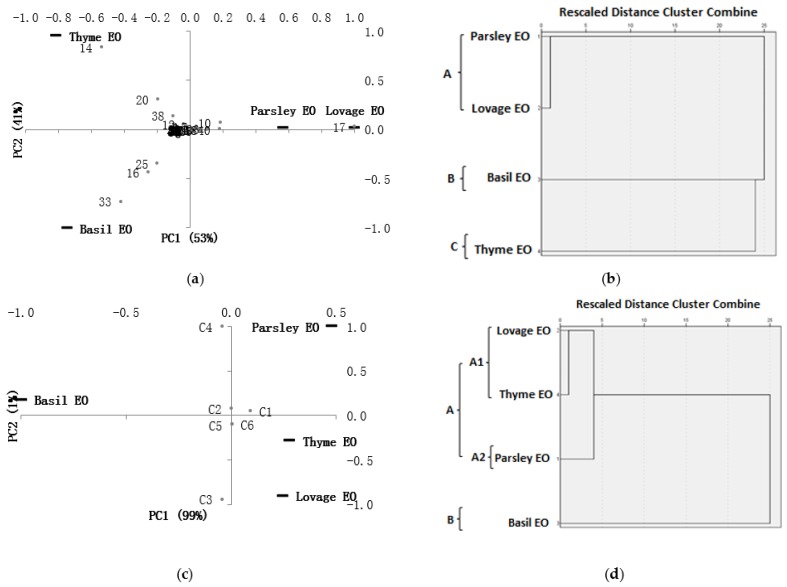
PCA and HCA performed on essential oil volatile compounds and their chemical classes. (**a**) The PCA biplot of essential oils based on their individual volatile compounds; (**b**) HCA dendrogram of essential oils based on their individual volatile compounds; (**c**) The PCA biplot of essential oils based on their chemical compound classes; (**d**) HCA dendrogram of essential oils based on their chemical compound classes. The numbers of chemical compounds are presented in Table 1; C1—monoterpene hydrocarbons; C2—sesquiterpene hydrocarbons; C3—oxygenated monoterpenes; C4—phenylpropanoids; C5—other compounds, and C6—unidentified compounds.

Chemical PCA and HCA analysis of all compounds. The chemical constituents of parsley, lovage, basil and thyme EOs were subjected to principal component analysis (PCA) in order to better visualize and identify their grouping trends. The PCA biplot (Figure 1a) indicates high similarities among parsley and lovage EOs (upper-right plot), characterized by β-phellandrene (compound No. **17** in Table 1 and Figure 1a) and β-myrcene (No. **10**). The first principal component (PC1) explained 53% of the total variance while the second one (PC2) showed a further 41%. Basil and thyme EOs instead were positioned very distinctly based on their characteristics constituents: Estragole (No. **33**), 1,8-cineole (No. **16**), and linalool acetate (No. **25**) for basil EO (bottom-left plot), respectively *p*-cymene (No. **14**), γ-terpinene (No. **20**), and thymol (No. **38**) for thyme EO (upper-left plot). Similar to PCA, the hierarchical cluster analysis (HCA) based on the Euclidean distance between groups (Figure 1b) indicated three groups (A, B, and C) of essential oils; parsley and lovage EOs formed one group (group A), while basil and thyme EOs were individual distinctly separated in group B and group C, respectively.

Chemical PCA and HCA analyses of compound class: In this case, PCA and HCA (Figure 1c,d) revealed interesting and somehow surprising results with lovage and thyme EOs forming 1st sub-group (A1) and parsley EO the 2nd one (A2) of the same cluster (group A). Basil EO formed another cluster (group B). The PCA biplot of essential oils based on their chemical compound classes (Figure 1c) showed a great chemical resemblance between lovage and thyme EOs (down-right plot), characterized by similar ratios of monoterpene hydrocarbons-C1 and oxygenated monoterpenes-C3 (83.6% C1 and 15.6% C3, respectively 83.5% C1 and 9.7% C3). PC1 horizontal axis explained 99% while PC2 vertical axis explained 1% of the total variance. On the same side of the axes (upper-right plot), parsley EO revealed higher ratio C1:C3 than thyme and lovage EOs such as 97.2% C1 and 0.9% C3. On the other side of the axes (upper-left plot), basil EO showed a much lower level of monoterpene hydrocarbons-C1 (10.7%) and higher levels of oxygenated monoterpenes-C3 (46.4%) and phenylpropanoids-C4 (41.2%) than parsley, lovage, and thyme EOs.

**Figure 2:**
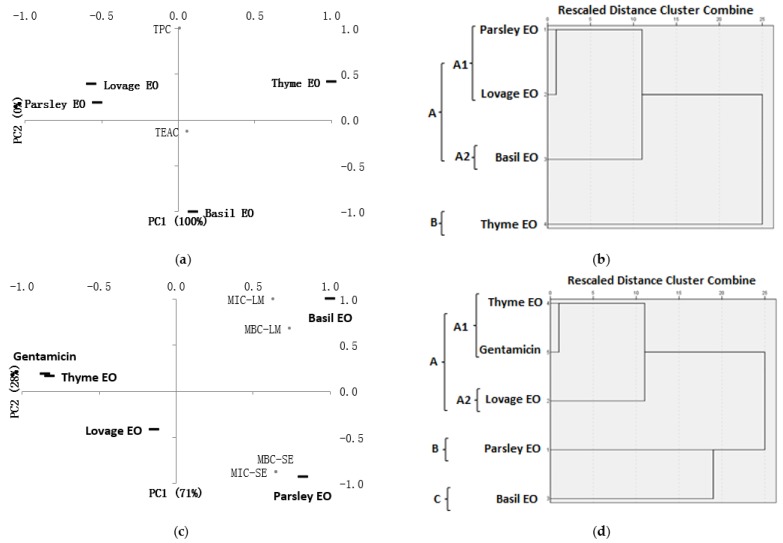
PCA and HCA performed on TPC and TEAC of essential oils. (**a**) The PCA biplot of essential oils based on their TPC and TEAC; (**b**) HCA dendrogram of essential oils based on their TPC and TEAC; (**c**) The PCA biplot of essential oils based on their MICs and MBCs; (**d**) HCA dendrogram of essential oils based on their MICs and MBCs. TPC—total phenolic content; TEAC—Trolox equivalent antioxidant capacity; MIC—minimum inhibitory concentration; MBC—minimum bactericidal concentration; LM—*L. monocytogenes*; SE—*S. enteritidis*.

PCA and HCA analysis of total phenolic content and antioxidant activity: It was interesting to note that performing PCA and HCA based only on total phenolic contents and antioxidant capacities (Figure 2a,b), a clearer image of essential oils characteristics has been revealed. The PCA biplot of essential oils based on their TPC and TEAC (Figure 2a) also confirmed a great similarity between parsley and lovage EOs (upper-left plot); these were clustered (Figure 2b) during PCA in the 1st sub-group (A1) of the first cluster (group A) with low TPC and weak antioxidant activity. PC1 horizontal axis explained 100% of the total variance. Basil EO (down-right of biplot in Figure 2a) formed the 2nd sub-group (A2) of the same cluster as was characterized by low TPC but moderate antioxidant activity. With the highest level of TPC and stronger antioxidant activity, thyme EO (upper-right plot in Figure 2b) was grouped into a second cluster (group B).

PCA and HCA analysis of MIC and MBC. PCA and HCA (Figure 2c,d) indicate high similarities among thyme EO and gentamicin (upper-left plot in Figure 2c; sub-group A1 in Figure 2d) that showed the stronger antibacterial activity against both tested strains. Lovage EO was plotted close to them but in the bottom-left quarter of the chart as it exhibited moderate antibacterial activity; it formed the 2nd sub-group (A2) of the same cluster (group A). PC1 horizontal axis explained 71% of the total variance while the vertical axis explained a further 28%. Other two distinct clusters were formed by parsley (group B) and basil EOs (group C), each one showing different antibacterial properties, parsley EO (bottom-right plot in Figure 2c) being less efficient against *S. enteridis* while basil EO (upper-right plot in Figure 2c) against *L. monocytogenes*.

## 4. Materials and Methods

### 4.1. Experimental Design

The current study was conducted to determine the chemical composition of four essential oils extracted from herbs belonging to the families Apiaceae (parsley and lovage) and Lamiaceae (basil and thyme), their total phenolic content (TPC), antioxidant activity (TEAC), as well as minimum inhibitory and bactericidal concentrations (MIC/MBC) against two of the most common bacteria responsible for food poisoning (*Salmonella enteritidis* and *Listeria monocytogenes*).

### 4.2. Plant Materials and Essential Oils Extraction

Commercially available leaves of parsley, lovage, basil, and thyme, in the dried state, were purchased from a company that markets food ingredients (Solina Group, Alba Iulia, Romania, http://www.solina-group.ro). Essential oils were obtained by hydrodistillation, dried leaves (50 g) of each plant material being extracted for 3 h with 750 mL distilled water in a Clevenger-type apparatus (S.C. Energo-Metr S.R.L., Odorheiu Secuiesc, Romania). The essential oils were dried over anhydrous sodium sulphate and stored at 4 °C until analysis. The extraction yield was calculated as the volume of essential oil (mL) per dried leaves weight (g) and multiplied by 100 [8].

### 4.3. ITEX/GC-MS Analysis of Volatile Constituents

Qualitative ITEX/GC-MS analysis of the volatile compounds was performed using an analytical technique based on in-tube extraction (ITEX), as previously reported by our group [25]. Sample of 1 µL volume was measured into a sealed-cap headspace vial (20 mL) and maintained on continuous agitation at 50 °C for 10 min. The adsorption (5 strokes) of volatile compounds from the gaseous phase of the essential oil was performed by a Combi PAL AOC-5000 autosampler (CTC Analytics, Zwingen, Switzerland) with a headspace syringe ITEX-II equipped with a microtrap (ITEX-2TrapTXTA, Tenax TA 80/100 mesh, Switzerland). The analytes were released by thermal desorption into the injection port of the GC-MSQP2010 system (Shimadzu, Kyoto, Japan); the microtrap was then flash-heated with N_2_.

A Zebron ZB-5 ms capillary column (30 m × 0.25 mm i.d. × 0.25 μm film thickness; Phenomenex, Torrance, CA, USA) was used for the chromatographic separation of volatile compounds. The column oven temperature program was set as follows: From 40 °C (kept at this temperature for 2 min) to 160 °C at 4 °C/min, then raised to 240 °C at 15 °C/min (kept at this temperature for 5 min). The ion source, injector, and interface temperatures were set at 250 °C. Helium was used as carrier gas, the constant flow rate being 1 mL/min. The split ratios were 1:200 for parsley EO, 1:250 for lovage and basil EOs, and 1:300 for thyme EO. The MS mode used was Electron Impact (EI) ionization, while a mass range from 40 to 650 *m/z* was scanned.

The volatile compounds were tentatively identified by comparing their mass spectra with those in the NIST27 and NIST147 libraries (considering a minimum similarity of 85%) and by retention indices obtained from www.pherobase.com [27] or www.flavornet.org [28] (for columns with a similar stationary phase to ZB-5 ms). The results were expressed as a percentage of the total peaks area.

### 4.4. Determination of Total Phenolic Content

The total phenolic content was measured using the method described by Socaci et al., 2014 [29]. One hundred microliters of test sample (dilution of essential oil with methanol—parsley EO:MeOH, 1:50 (*v*/*v*); lovage EO:MeOH, 1:50 (*v*/*v*); basil EO:MeOH, 1:50 (*v*/*v*); and thyme EO:MeOH, 1:250 (*v*/*v*)) were transferred into a 16 mL glass bottle with a rubber stopper. Then, 6 mL of distilled water and 0.5 mL of 2 N Folin-Ciocalteu phenol reagent were added and immediately vortexed (Vortex V-1 Plus, Biosan Ltd., Riga, Latvia). After 4 min, 1.5 mL of 0.71 M sodium carbonate aqueous solution and 1.9 mL distilled water were added to the mixture. The incubation of the test sample was carried out in the dark at room temperature for 2 h. The absorbance value was read at 725 nm against blank sample using a double beam UV-VIS spectrophotometer (PharmaSpec UV-1700, Shimadzu, Kyoto, Japan). The blank sample was prepared with methanol and treated identically to the test sample. Three readings per sample were taken. The result was expressed in mg gallic acid equivalents (GAE) 100 µL^−1^ EO.

### 4.5. Determination of Antioxidant Activity

The Trolox equivalent antioxidant capacity assay was used to measure the antioxidant activity of essential oils; it was performed according to the method described by Thaipong et al., 2006 [30]. The stock solution was prepared by mixing equal volumes of 7.4 mM ABTS aqueous solution and 2.6 mM potassium persulfate aqueous solution and allowing them to react for 12 h at room temperature in the dark. The working solution was prepared by mixing 1 mL of stock solution with 60 mL of methanol to obtain an absorbance of 1.1 ± 0.02 units at 734 nm. One hundred and fifty microliters of test sample (dilution of essential oil with methanol—parsley EO:MeOH, 1:100 (*v*/*v*); lovage EO:MeOH, 1:100 (*v*/*v*); basil EO:MeOH, 1:300 (*v*/*v*); and thyme EO:MeOH, 1:500 (*v*/*v*)) were transferred into a 16 mL glass bottle with a rubber stopper, then 2850 μL of ABTS working solution were added and vortexed. The incubation of the test sample was carried out in the dark, at room temperature for 2 h. The absorbance value was read at 734 nm against methanol using a double beam UV-VIS spectrophotometer (PharmaSpec UV-1700, Shimadzu, Kyoto, Japan). The blank sample was prepared with methanol and treated identically to the test sample. Three readings per sample were taken. The absorbance value of the test sample is extracted from the absorbance value of the blank sample; the final result was expressed in µM Trolox equivalent (TE) mL^−1^ EO.

### 4.6. Bacterial Strains

The following microorganisms were tested: *Salmonella enteritidis* (ATCC 13076, Microbiologics Inc., St. Cloud, MN, USA) and *Listeria monocytogenes* (ATCC 19114, Microbiologics Inc., St. Cloud, MN, USA). Each strain was grown into a test tube containing 10 mL sterile nutrient broth (Oxoid Ltd., Basingstoke, Hampshire, England) at 37 °C for 24 h in the case of *S. enteritidis* and at 37 °C for 48 h in the case of *L. monocytogenes*. The purity of the inoculum was confirmed by plating on appropriate selective media and microscopic examination of the Gram-stained smear (Optika microscope, B-252, M.A.D. Apparecchiature Scientifiche, Milan, Italy). A loopful of inoculum was transferred by streaking onto a selective medium: XLD agar (Oxoid Ltd., Basingstoke, Hampshire, England) for *S. enteritidis* and Palcam agar base (Oxoid Ltd., Basingstoke, Hampshire, England) with added Palcam selective supplement for *L. monocytogenes*. Plates were incubated at 37 °C for 24 h in the case of *S. enteritidis* and at 37 °C for 48 h in the case of *L. monocytogenes*. Bacterial morphology was confirmed by optical microscopy. Several colonies were collected with a sterile inoculating loop, transferred into sterile saline solution (8.5 g L^−1^), and adjusted to match the turbidity of a McFarland 0.5 standard (1.5 × 10^8^ CFU mL^−1^) [31]. Then, three *serial* 10-fold *dilutions* (10^7^, 10^6^, and 10^5^ CFU mL^−1^) were prepared using the sterile saline solution as diluent.

### 4.7. Minimum Inhibitory Concentration Test (MIC)

The test was performed according to the method described by Semeniuc et al., 2017 [8]. Eight parts 50% ethanol solution and one part Tween 80 were mixed with one part of the essential oil [32]. Sterile nutrient broth (100 µL) and diluted essential oil (100 µL) were added into the first well of a 96-well microtiter plate. Serial 11-fold dilutions were obtained by pipetting 100 µL from well to well (on row), while 100 µL were discarded from the last well of the row. Next, a volume of 10 µL inoculum (1.5 × 10^5^ CFU mL^−1^) was added to each well. Concentrations ranging from 0.01 to 47.62 µL EO mL^−1^ were thus reached. For the negative control, eight parts 50% ethanol and one part Tween 80 were mixed with one part of the saline solution, while gentamicin (0.4 mg mL^−1^ in saline solution) was considered the positive control. Microplates were incubated at 37 °C for 21 h in the case of *S. enteritidis* and at 37 °C for 45 h in the case of *L. monocytogenes*. A volume of 20 μL resazurin aqueous solution (0.2 mg mL^−1^) was added to each well. Microplates were subsequently incubated at 37 °C for 2 h in the case of *S. enteritidis* and at 37 °C for 23 h in the case of *L. monocytogenes*. The lowest concentration that retained the blue color was considered the concentration that completely inhibited bacterial growth (MIC). Three replicates were run for each essential oil. The result was expressed in µL EO mL^−1^, respectively µg gentamicin (GE) mL^−1^.

### 4.8. Minimum Bactericidal Concentration Test (MBC)

To determine the *minimum bactericidal concentration*, 10 µL of the dilution representing the MIC and the more concentrated two dilutions were plated on the appropriate selective media (XLD agar for *S. enteritidis* and Palcam agar base with added Palcam selective supplement for *L. monocytogenes*) using a Drigalski spatula and incubated for 48 h at 37 °C. A colony counter (Colony Star 8500, Funke Gerber, Berlin, Germany) was used to determine the relative number of bacterial colonies. The lowest concentration of the antimicrobial agent causing negative growth (fewer than three colonies) was considered to be the concentration that completely killed bacteria [32]. The result was expressed in µL EO mL^−1^.

### 4.9. Statistical Analysis

Hierarchical clustering (HCA) and correlation analysis were carried out using IBM SPSS Statistics software, version 19.0 (IBM Corp., Armonk, New York, NY, USA). Cluster analysis was run using the complete linkage method. Correlation analysis between was performed via Pearson’s correlation. Principal component analysis (PCA) was carried out using Unscrambler software, version 9.7 (CAMO Software AS, Oslo, Norway).

## 5. Conclusions

Monoterpenes hydrocarbons were the most abundant chemical compounds in parsley, lovage, and thyme EOs, while in basil, oxygenated monoterpenes and phenylpropanoids prevailed. Regarding the antibacterial activity, all tested essential oils showed inhibitory and bactericidal effects on both bacteria; most of them were more effective against *L. monocytogenes* (ATCC 19114) but basil EO was more effective against *S. enteritidis* (ATCC 13076). The highest amount of total phenolics and antioxidant capacity were found in thyme EO; this essential oil also exhibited the stronger antibacterial activity. Parsley EO instead had a low total phenolic content (TPC), weak antioxidant activity, and weak antibacterial activity against *S. enteritidis* (ATCC 13076); lovage EO by low TPC, weak antioxidant activity, and moderate antibacterial activity; basil EO by low TPC, moderate antioxidant activity, and weak antibacterial activity against *L. monocytogenes* (ATCC 19114)*.*

## Figures and Tables

**Table 1:** Volatile compounds detected in essential oils.

Crt. No.	Compound	CC	RT (min)	RC (%)			
				Parsley EO	Lovage EO	Basil EO	Thyme EO
**1**	Methyl 2-methylbutanoate	C5	4.763	-	-	-	2.34
**2**	Hexanal	C5	5.346	0.42	0.50	-	-
**3**	4-Nonene	C5	8.550	0.07	-	-	-
**4**	α-Thujene	C1	9.666	0.05	0.26	0.19	2.04
**5**	**α-Pinene**	C1	9.924	**6.49**	3.63	1.92	3.81
**6**	Camphene	C1	10.565	-	0.33	0.32	1.68
**7**	Sabinene	C1	11.502	0.42	1.39	1.06	-
**8**	β-Pinene	C1	11.668	2.78	0.89	2.57	0.58
**9**	2-Octen-1-ol, (E)-	C5	11.835	-	-	-	0.32
**10**	**β-Myrcene**	C1	12.185	**12.71**	**11.36**	1.37	**5.41**
**11**	α-Phellandrene	C1	12.817	2.93	1.53	-	0.35
**12**	n.i.	C6	13.269	0.09	-	-	3.50
**13**	α-Terpinene	C1	13.289	-	-	0.29	-
**14**	***p*-Cymene**	C1	13.594	1.65	0.99	0.43	**49.81**
**15**	**Limonene**	C1	13.755	**12.46**	-	1.81	1.11
**16**	**1,8-Cineole**	C3	13.904	-	-	**24.30**	0.30
**17**	**β-Phellandrene**	C1	13.908	**32.44**	**53.89**	-	-
**18**	**trans-β-Ocimene**	C1	14.064	0.13	**7.00**	-	-
**19**	cis-β-Ocimene	C1	14.486	0.30	-	0.36	-
**20**	**γ-Terpinene**	C1	14.956	0.68	0.78	0.41	**18.56**
**21**	n.i.	C6	15.469	-	-	0.09	-
**22**	Terpinolene	C1	16.023	3.76	1.53	-	0.10
**23**	Fenchone	C3	16.190	-	-	0.22	-
**24**	**2,6-Dimethylstyrene**	C1	16.226	**6.43**	-	-	-
**25**	**Linalool acetate**	C3	16.644	0.08	0.81	**19.80**	0.56
**26**	**1,3,8-** ***p*** **-Menthatriene**	C1	17.127	**13.93**	-	-	-
**27**	Fenchol	C3	17.429	-	-	0.34	-
**28**	Camphor	C3	18.530	-	-	0.24	-
**29**	Menthone	C3	18.861	0.06	-	-	-
**30**	Isoborneol	C3	19.506	-	-	-	0.11
**31**	trans-4-Thujanol	C3	19.846	-	-	0.54	-
**32**	α-Terpineol	C3	20.419	-	1.96	0.48	-
**33**	**Estragole**	**C4**	20.518	-	-	**40.93**	-
**34**	Anisole	C4	21.690	-	-	-	0.19
**35**	Carvone	C3	22.236	0.52	0.13	-	-
**36**	Bornyl acetate	C3	23.767	-	-	0.14	-
**37**	Anethole	C4	23.807	0.79	0.30	-	-
**38**	**Thymol**	C3	23.951	-	-	0.29	**8.72**
**39**	Phenol, 2-ethyl-4,5-dimethyl-	C5	24.240	-	-	-	0.35
**40**	**β-Terpinyl acetate**	C3	25.973	0.24	**12.73**	-	-
**41**	n.i.	C6	27.027	0.13	-	-	-
**42**	Eugenol methyl ether	C4	27.856	-	-	0.29	-
**43**	Caryophyllene	C2	28.583	0.43	-	0.70	0.16
**44**	α-Bergamotene	C2	29.003	-	-	0.92	-
	**TOTAL**			**100.0**	**100.0**	**100.0**	**100.0**

CC—chemical class; C1—monoterpene hydrocarbons; C2—sesquiterpene hydrocarbons; C3—oxygenated monoterpenes; C4—phenylpropanoids; C5—other compounds; C6—unidentified compounds; RT—retention time; RC—relative content of peak area; n.i.: not identified.

**Table 2:** Total phenolic contents (TPC) and antioxidant activities (TEAC) of essential oils.

Essential Oil	TPC (mg GAE 100 µL^−1^ EO)	TEAC (µM TE mL^−1^ EO)
Parsley EO	2.2 ± 0.05	39.4 ± 0.02
Lovage EO	3.0 ± 0.15	35.9 ± 0.27
Basil EO	2.9 ± 0.13	105.0 ± 1.09
Thyme EO	22.5 ± 1.69	197.0 ± 0.23

Values are results of three replicates.

**Table 3:** Minimum inhibitory (MIC) and minimum bactericidal (MBC) concentrations of essential oils.

Essential Oil	*S. Enteritidis*/ATCC 13076		*L. Monocytogenes*/ATCC 19114	
Parsley EO	22.68 ± 0.0	22.68 ± 0.0	5.14 ± 0.0	10.80 ± 0.0
Lovage EO	10.80 ± 0.0	10.80 ± 0.0	2.45 ± 0.0	2.45 ± 0.0
Basil EO	10.80 ± 0.0	10.80 ± 0.0	22.68 ± 0.0	22.68 ± 0.0
Thyme EO	0.56 ± 0.0	0.56 ± 0.0	0.13 ± 0.0	0.13 ± 0.0

Values are results of three replicates. Gentamicin’s MBCs, which were identical to the MICs, were 0.11 ± 0.0 for *S. enteritidis* (ATCC 13076) and 0.05 ± 0.0 µg GE mL^−1^ for *L. monocytogenes* (ATCC 19114).
